# Calling for more public health resources for women: findings from the burden of low back pain in China

**DOI:** 10.3389/fpubh.2025.1562760

**Published:** 2025-11-18

**Authors:** Kaijie Shao, Yanping Qu, Jingjing Fan, Xiaoyue Zhu, Xiaoxiao Hu, Yanhong Ma, Huaichun Yang

**Affiliations:** 1 School of Exercise and Health, Shanghai University of Sport, Shanghai, China; 2 Department of Rehabilitation Medicine, Shanghai Sixth People's Hospital Affiliated to Shanghai Jiao Tong University School of Medicine, Shanghai, China; 3 Department of Rehabilitation Medicine, Shanghai Eighth People's Hospital, Shanghai, China; 4 Shanghai Key Laboratory of Sleep Disordered Breathing, Department of Otolaryngology Head and Neck Surgery, Shanghai Sixth People's Hospital Affiliated to Shanghai Jiao Tong University School of Medicine, Shanghai, China; 5 Department of Radiology, The First Affiliated Hospital, Sun Yat-sen University, Guangzhou, Guangdong, China

**Keywords:** low back pain, China, female, joinpoint regression, age-period-cohort analysis, ARIMA model

## Abstract

**Background:**

To reveal the burden and progression of low back pain in China from perspectives such as gender and age using data from the Global Burden of Disease 2019 (GBD 2019).

**Methods:**

The data we used are all from the Global Burden of Disease dataset. We calculated Annual Percentage Change (APC) and Average Annual Percentage Change (AAPC) from 1990 to 2019 by Joinpoint regression analysis. Meanwhile, the independent effects of age, period and cohort were estimated using Age-Period-Cohort analysis. Autoregressive integrated moving average (ARIMA) model was used to predict the trend of LBP prevalence in the next decade.

**Results:**

From 1990 to 2019, age-standardized prevalence rates (ASPR) and incidence rates (ASIR) of low back pain in China declined significantly, yet the total prevalence and incidence continued to rise, with higher rates in women. And we predict it will continue to rise in the next decade. Disability-Adjusted Life Years (DALYs), Years Lived with Disability (YLDs) were consistently higher in women, while age-standardized YLDs and DALYs rates decreased, though total YLDs and DALYs continued to rise.

**Conclusion:**

Low back pain is a substantial public health burden in China. Notably, women face a higher risk and warrant special attention.

## 1 Background

Low back pain (LBP) is a major contributor to global disability, with its burden magnified in aging populations (1). LBP is a very common symptom that occurs in all age groups and in countries at all income levels (2). It has substantial impacts on daily life and work productivity, and the high cost and prevalence of LBP place a heavy burden on society and healthcare systems. While international studies identify elevated LBP prevalence among women, linked to biological vulnerabilities, occupational exposures, and psychosocial stressors (3, 4), China’s distinct epidemiological landscape demands urgent scrutiny.

China is one of the most populous countries in the world, with an estimated population of approximately 1.4 billion in 2019 (source: https://countrymeters.info/cn/China); however, there is a lack of comprehensive studies on the prevalence of LBP in China across gender, age, period, and cohort dimensions. Previous epidemiological studies on LBP have been limited in their in-depth exploration of gender differences. The Global Burden of Disease study systematically quantified health losses by age, sex, year, and geographic location for more than 350 diseases, and the data are continually updated (5). The results of GBD 2019 now supersede previous GBD data. We presented the long-term trends in the burden of LBP in China from 1990 to 2019, including incidence, prevalence, Years Lived with Disability (YLDs), Years of Life Lost (YLLs), and Disability-Adjusted Life Years (DALYs). Distinguishing the relative contributions of period effects and cohort effects to the overall temporal trends can help to determine the success of early policy interventions and to set future targets (6). LBP’s high prevalence and burden necessitate updating its epidemiological data.

This study aims to assess the long-term trends of LBP in China (1990–2019) and predict its burden in 2020–2029. The study also explored the gender differences in low back pain and provided reference suggestions for gender equality strategies in addressing the disease burden.

## 2 Methods

### 2.1 Overview

This study systematically quantified the burden of LBP in China from 1990 to 2019 and projected trends through 2029 using data from the GBD 2019. Spanning three decades across all age groups (0–94 years), the analysis employed sex-specific stratification to reveal disparities in prevalence, incidence, DALYs, and YLDs between males and females. By integrating age-period-cohort modeling and autoregressive integrated moving average (ARIMA) forecasting, the research dynamically examined the combined impacts of population aging, socioeconomic transformation, and healthcare policy evolution on LBP trends. The Ninth Edition (ICD-9) and Tenth Edition (ICD-10) of International Classification of Diseases were used to classify LBP (5).

Methodologically, the study adopted Joinpoint regression to identify nonlinear trends (calculating Annual Percentage Change [APC] and Average Annual Percentage Change, [AAPC]). APC model was to disentangle age effects, period effects, and birth cohort effects. ARIMA model was to incorporate demographic and socioeconomic development parameters for sex-stratified projections. Data sources included validated GBD 2019 databases and national census records, analyzed using Joinpoint 5.0.2 and R 4.3.3 (R software), with full compliance to GATHER guidelines.

### 2.2 Data sources

LBP data from 1990 to 2019 were sourced from the WHO-supported GBD project, accessible via its health data platform.^1^ In China, GBD data were sourced from national censuses, disease surveillance, maternal and child health systems, and chronic disease monitoring (7). We extracted data on LBP in China (1990–2019) from GBD 2019, and analyzed prevalence, incidence, YLLs, YLDs, and DALYs for approximately 30 years.

### 2.3 Statistical analysis

#### 2.3.1 Joinpoint regression analysis

To address the potential nonlinear trends in LBP burden influenced by socioeconomic transitions and policy interventions over three decades, we used Joinpoint regression (Version 5.0.2, May 2023, Statistical Methodology and Applications Branch, Surveillance Research Program, National Cancer Institute) to analyze temporal trends in LBP prevalence in China (1990–2019) and identify critical inflection points of trend changes, using the Monte Carlo Permutation Test to validate significant changes. This method employed segmental regression to divide longitudinal changes into discrete intervals, overcoming the limitation of assuming uniform trends in conventional linear models (8). The Monte Carlo Permutation Test (10,000 simulations) was applied to determine the optimal number of segments, ensuring data-driven identification of trend turning points (e.g., post-policy implementation periods) while controlling for type I errors. We calculated the APC and AAPC, with 95% confidence intervals (CIs), to quantify sex-specific progression rates and contextualize China’s progress against global benchmarks. This approach is suitable for dissecting complex public health trajectories shaped by aging, occupational shifts, and healthcare reforms (9).

#### 2.3.2 Age-period-cohort analysis

To disentangle the complex interplay of biological aging, temporal policy shifts, and generational risk exposures underlying LBP disparities, the APC model was used to assess the impact of age, period, and cohort effects on health outcomes. The age effect describes differences in the incidence of LBP by age, critical for identifying high-risk age groups in China’s aging population; the period effect reflects the influence of social factors on the incidence of LBP, reflecting population-wide influences such as healthcare reforms or occupational safety regulations (e.g., post-2003 rural health insurance expansion); and the cohort effect refers to differences in exposure to risk factors among people born in different generations, which may drive persistent gender disparities (10). Age groups were categorized from 0–4 to 90–94 years, with 5-year intervals spanning 20 cohorts. An intrinsic estimator (IE) method based on Poisson log-linear regression was integrated into the APC model to estimate the net effects of the three dimensions (11). Bias, Bayesian information criterion (BIC), and Akaike information criterion (AIC) were used to assess the degree of model fit. Relative risk (RR) was used to interpret the estimated parameters of the model, enabling direct comparisons of age-specific vulnerabilities. The analysis was performed using the APC Analysis web tool developed by National Cancer Institute (12).

#### 2.3.3 ARIMA model

To project the long-term burden of LBP under China’s accelerating population aging and evolving healthcare policies, the ARIMA model was used to predict future trends in the incidence of LBP. This method is suited for non-stationary epidemiological time series where trends and external shocks (e.g., policy interventions) introduce complex temporal dependencies. The model expression was ARIMA (p, d, q), which integrates autoregressive order (p), differencing degree (d), and moving average order (q) to adaptively capture both short-term fluctuations and secular trends in LBP incidence. Time series stationarity was assessed using the Augmented Dickey-Fuller (ADF) test. For non-smooth series, the smoothness was achieved by the appropriate number of differencing treatments. Autocorrelation function (ACF) and partial autocorrelation function (PACF) plots were utilized to guide the selection of ARIMA model parameters. To optimize model parsimony and generalizability, model selection and parameter optimization were conducted via the auto.arima function in R, based on AIC and BIC criteria. This automated approach reduced subjective bias in parameter tuning while prioritizing models balancing fit quality and complexity. Model residuals were analyzed to verify validity. ACF plots and Ljung-Box tests were used to assess residual randomness, ensuring that the model effectively captured the key temporal patterns and validated its capacity to extract key temporal signals, thereby supporting robust gender-specific projections through 2029. The analysis was performed using the R software (version 4.3.3).

## 3 Results

### 3.1 Descriptive analysis

**Figure 1:**
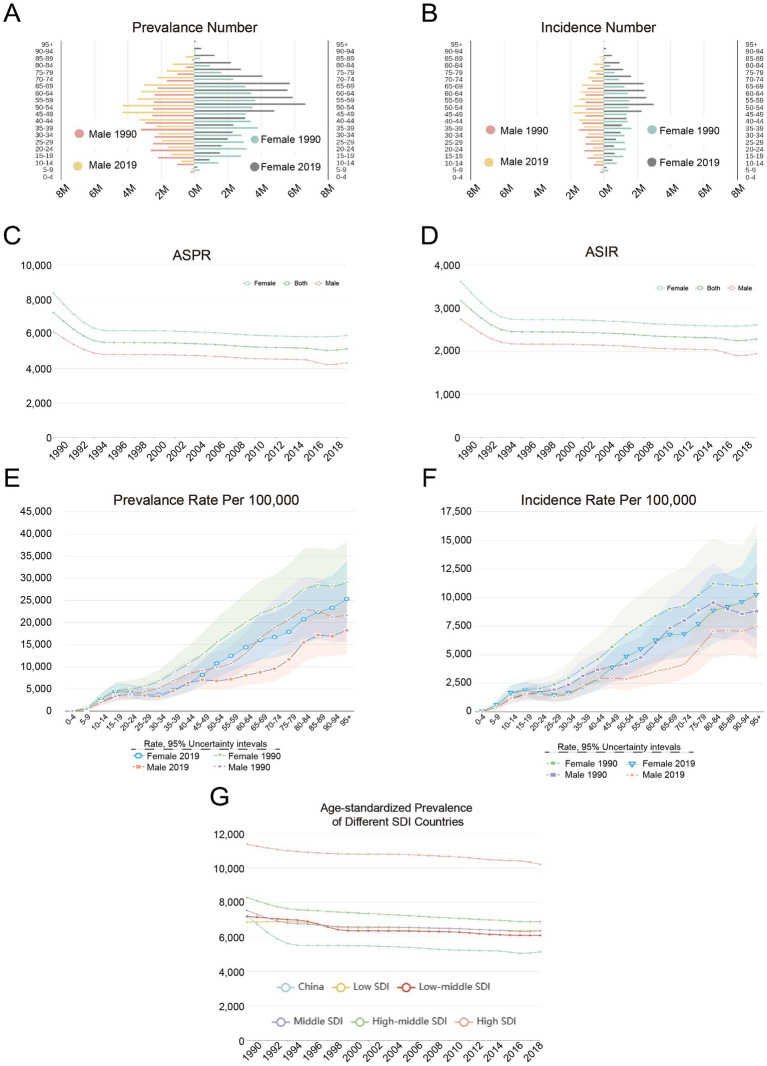
Number and rate of prevalence and incidence, and age-standardized prevalence and incidence rates of LBP in China, 1990–2019. **(A)** Prevalence number. **(B)** Incidence number. **(C)** Age-standardized prevalence rate (ASPR). **(D)** Age-standardized incidence rate (ASIR). **(E)** Prevalence rate per 100,000. **(F)** Incidence rate per 100,000. **(G)** Age-standardized prevalence of different SDI countries. UI, uncertainty interval, representing the 95% confidence range for the estimates. SDI, socio-demographic index.

In 2019, there were a total of 913,394,32 (95% uncertainty interval, UI: 805,279,92–104,119,887) cases of LBP in China, of which 375,675,31 (95% UI: 330,239,75–428,588,92) were men and 537,719,00 (95% UI: 474,561,81–616,182,65) were women (Figure 1A). Gender analysis showed that the age-standardized prevalence rate in females was higher than that of males. Between 1990 and 2019, the age-standardized prevalence rate in China decreased from 7245.29 (95% UI: 6390.00–8170.48) to 5134.73 (95% UI: 4548.46–5786.97) (Figure 1C), indicating a decreasing trend. However, the total number of patients with LBP was increasing due to population growth and aging (Figures 1A,B). And we compared the LBP burden in China with other countries based on Socio-demographic Indices (SDI), which reflect a country’s social development, including education level, economic status, and fertility rates (13). Age-standardized prevalence rates across all SDI countries were declining, with China’s rate remaining relatively low (Figure 1G). The age-standardized incidence rate in China decreased from 3,174.26 (95% UI: 2,806.28–3,598.26) to 2,280.67 (95% UI, 2,017.18–2,572.62) per 100,000 people (Figure 1D); when comparing gender-specific prevalence and incidence rates between 1990 and 2019, both metrics were consistently higher in females than in males, whereas the 2019 data for both males and females were lower than the 1990 data (Figures 1E,F). The specific data on the prevalence, incidence, DALYs, YLDs, and YLLs of LBP in 2019 are shown in Table 1.

**Table 1:** The specific data on the prevalence, incidence, DALYs, YLDs, and YLLs of LBP in 2019.

Measure	All-ages cases			Age-standardized rates per 100,000 people		
	Both	Male	Female	Both	Male	Female
Prevalence	91339432.23 (80527992.89, 104119887.7)	37567531.94 (33023975.81, 42858892.12)	53771900.29 (47456181.81, 61618265.38)	5134.73 (4548.463, 5786.97)	4328.76 (3824.27, 4898.42)	5915.76 (5240.23, 6670.08)
Incidence	40158356.95 (35369447.69, 45809146.32)	16624267.30 (14600948.09, 19029395.81)	23534089.65 (20795062.81, 26827166.12)	2280.67 (2017.18, 2572.62)	1941.68 (1711.82, 2201.89)	2611.58 (2314.86, 2947.58)
DALYs	10334104.360 (7329879.12, 14004811.71)	4307306.06 (3017048.01, 5857231.41)	6026798.30 (4270860.45, 8179084.44)	579.05 (411.61, 778.09)	493.39 (348.96, 660.80)	662.76 (471.37, 884.0)
YLDs	10334104.36 (7329879.14, 14004811.71)	4307306.06 (3017048.01, 5857231.41)	6026798.30 (4270860.45, 8179084.44)	579.05 (411.61, 778.09)	493.39 (348.96, 660.80)	662.76 (471.37, 884.0)
YLLs	228407693.88 (197155840.99, 260733993.43)	143933021.08 (117983346.03, 173944767.88)	84474672.80 (70148482.97, 101038776.61)	13346.98 (11701.44, 14996.59)	17446.61 (14717.89, 20570.32)	9669.65 (8196.76, 11331.51)

DALYs, disability-adjusted life-years; YLDs, years lived with disability; YLLs, years of life lost; LBP, low back pain. Values are presented as mean with UI. UI, uncertainty interval, representing the 95% confidence range for the estimates. Age-standardized rates (ASRs) were calculated using the GBD 2019 standard population to control for confounding effects of population age structure, the specific calculation formulas can be found on the GBD Official Website.

### 3.2 Joinpoint regression analysis

**Figure 2:**
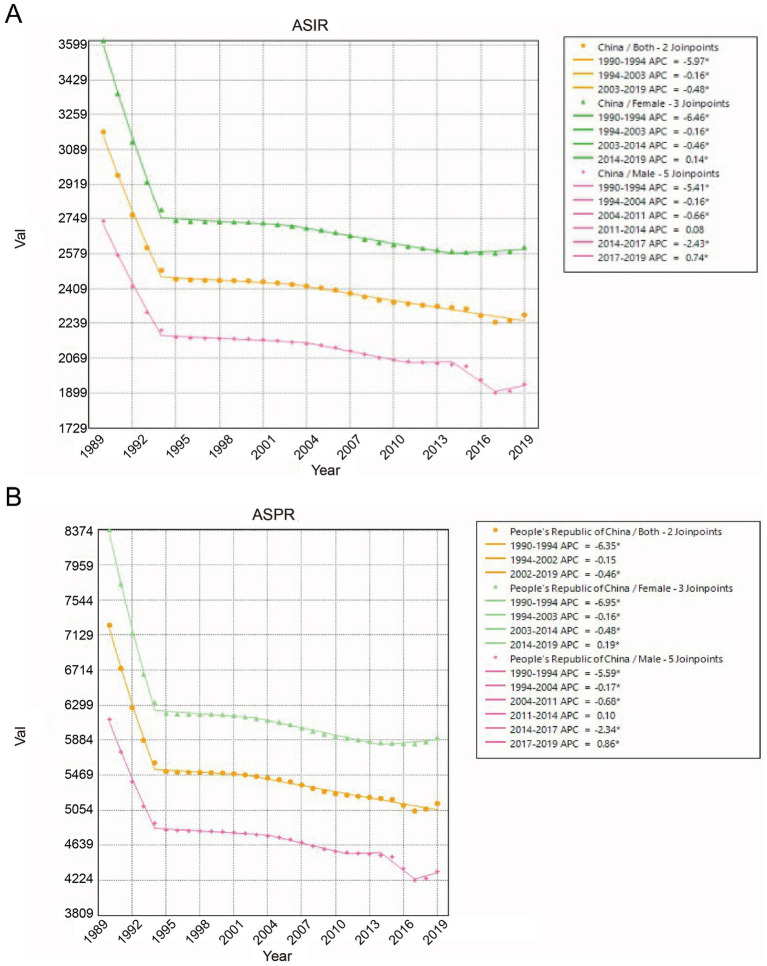
Joinpoint regression analysis of the sex-specific age-standardized incidence rates and prevalence rates for LBP in China from 1990 to 2019. **(A)** Age-standardized incidence rate (ASIR). **(B)** Age-standardized prevalence rate (ASPR). * Annual Percent Change (APC) was significantly different from zero at the alpha = 0.05 level.

From 1990 to 2019, the age-standardized incidence and prevalence of LBP in China showed a decreasing trend, and the magnitude of the decrease varied over time for men and women. As indicated by Joinpoint Regression Analysis, the APC for age-standardized incidence rate (ASIR) showed a decline in both males and females (Figure 2A). The APC for ASIR in males showed a significant decline between 1990 and 1994 (APC: −5.41*), followed by another significant decrease between 2014 and 2017. For the remaining periods, the APC for ASIR in males exhibited a steady downward trend. The peak of decline for females also occurred in 1990–1994 (APC: −6.46*), followed by no significant fluctuations in the subsequent phases, with a smooth downward trend. The results of Joinpoint Regression Analysis for age-standardized prevalence rate (ASPR) were essentially consistent with those of ASIR (Figure 2B). Table 2 shows the ASIR for LBP for the past 30 years and ASPR’s Joinpoint Regression Analysis specific data. The APC, AAPC, incidence, and prevalence values were lower in males than in females over the past 30 years.

**Table 2:** Joinpoint regression analysis: trends in age-standardized prevalence, incidence rates (per 100,000 persons) in China, 1990–2019.

Gender	ASPR			ASIR		
	Period	APC (95% CI)	AAPC (95% CI)	Period	APC (95% CI)	AAPC (95% CI)
Both	1990–1994	−6.35 (−6.78, −5.92)	−1.21(−1.29, −1.13)	1990–1994	−5.97 (−6.39, −5.56)	−1.16 (−1.23, −1.08)
	1994–2002	−0.15 (−0.34, 0.05)		1994–2003	−0.16 (−0.31, −0.01)	
	2002–2019	−0.46 (−0.51, −0.41)		2003–2019	−0.48 (−0.53, −0.42)	
Male	1990–1994	−5.59 (−5.75, −5.44)	−1.18 (−1.27, −1.10)	1990–1994	−5.41 (−5.56, −5.25)	−1.17* (−1.25, −1.09)
	1994–2004	−0.17 (−0.21, −0.12)		1994–2004	−0.16 (−0.21, −0.11)	
	2004–2011	−0.68 (−0.77, −0.59)		2004–2011	−0.66 (−0.75, −0.57)	
	2011–2014	0.10 (−0.43, 0.63)		2011–2014	0.08 (−0.45, 0.62)	
	2014–2017	−2.34 (−2.86, −1.82)		2014–2017	−2.43 (−2.95, −1.91)	
	2017–2019	0.86 (0.32, 1.41)		2017–2019	0.74 (0.20, 1.27)	
Female	1990–1994	−6.95 (−7.16, −6.74)	−1.19 (−1.23, −1.14)	1990–1994	−6.46 (−6.66, −6.27)	−1.12 (−1.16, −1.07)
	1994–2003	−0.16 (−0.24, −0.09)		1994–2003	−0.16 (−0.23, −0.09)	
	2003–2014	−0.4 8 (−0.54, −0.43)		2003–2014	−0.46 (−0.51, −0.41)	
	2014–2019	0.19 (0.04, 0.35)		2014–2019	0.14 (0.00, 0.29)	

Age-standardized prevalence rate, ASPR; APC, annual percent change; CI, confidence interval; AAPC, average annual percent change; ASIR, age-standardized incidence rate. The asterisk ‘*’ indicates the system’s preferred model.

### 3.3 The effects of age, period, and cohort on incidence rates

**Figure 3:**
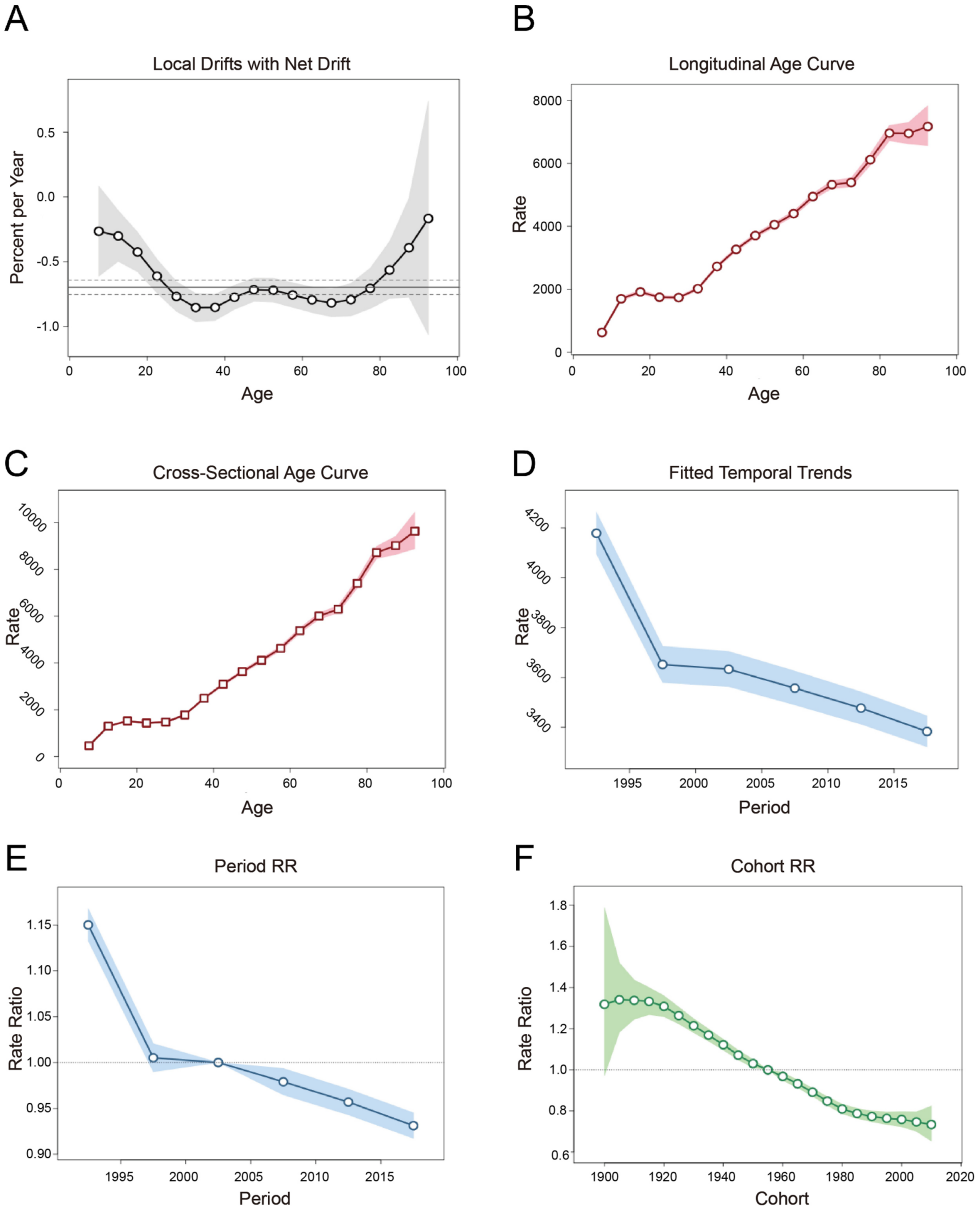
Analysis result of the incidence of LBP in China from 1990 to 2019 due to effects of age, period and cohort. **(A)** Local Drifts with Net Drift: analysis combining age-specific incidence trends (Local Drift) and overall age-adjusted trends (Net Drift), reflecting age-related variations and integrated period/cohort effects. **(B)** Longitudinal age curve: age-related incidence trends within the same birth cohort, capturing cumulative effects of aging. **(C)** Cross-Sectional Age Curve: Distribution of disease incidence across age groups within the same period, reflecting cross-sectional age-incidence relationships. **(D)** Fitted temporal trends: model-adjusted temporal trends in incidence rates, standardized to remove cohort bias. **(E)** Period RR (Relative risk): RR of disease incidence in a specific period compared to the reference period (rate ratio > 1 indicates higher risk). **(F)** Cohort RR: RR of disease incidence in a specific birth cohort compared to the reference cohort (rate ratio > 1 indicates higher risk).

From 1990 to 2019, LBP incidence in China showed an overall decline, particularly among individuals aged 22.5 to 77.5 years, with notable variations across age, period, and cohort. Net Drift [(%) per year] was the predominant outcome, representing the APC in expected age-adjusted rates over time, while accounting for both components of the trend attributable to period and cohort factors (14). The Net Drift for this outcome was −0.697, which was statistically significant and indicated a decreasing incidence rate. Figure 3A shows that incidence rates declined consistently across all age groups, with the most rapid decrease observed between 22.5 and 77.5 years. The Local Drift and Net Drift tests (*p*-value = 0.0033) indicated statistical significance, supporting the trend of declining incidence. Figures 3B,C show the results of the age effect analysis using longitudinal and cross-sectional age curves, where the longitudinal age curves represented the age-related prevalence of LBP, which increased with age in the same birth cohort. Figures 3D,E show period effects using Fitted Temporal Trends and Period RR, where rate ratio >1 indicated a higher morbidity risk compared to the reference period and rate ratio <1 indicated a lower risk. Specific data are provided in Table 3. Fitted Temporal Trends were the reference age-stratum morbidity rates corrected for cohort bias. The results showed a slow decline in incidence in more recent years compared with the previous period. Figure 3F represents the results of the birth cohort effect analysis using the Cohort RR. The results showed that compared with the reference cohort, those born before 1955 had a higher risk of morbidity, whereas those born after 1955 had a lower risk of morbidity, although all birth cohorts had reduced morbidity rates.

**Table 3:** Period RR of LBP in China.

Period	Rate ratio	CI Lo	CI Hi
1992.5	1.15	1.133	1.168
1997.5	1.005	0.99	1.021
2002.5	1	1	1
2007.5	0.979	0.965	0.994
2012.5	0.957	0.943	0.971
2017.5	0.931	0.917	0.945

RR, relative rate; LBP, low back pain; CI Lo, Lower limit of confidence interval; CI Hi, Higher limit of confidence interval.

### 3.4 Result of the forecasting model

According to the projections, the incidence of LBP in Chinese men and women would increase slightly between 2020 and 2029, with an expected increase to 2,291.5 per 100,000 for men and 2,941.5 per 100,000 for women.

**Figure 4:**
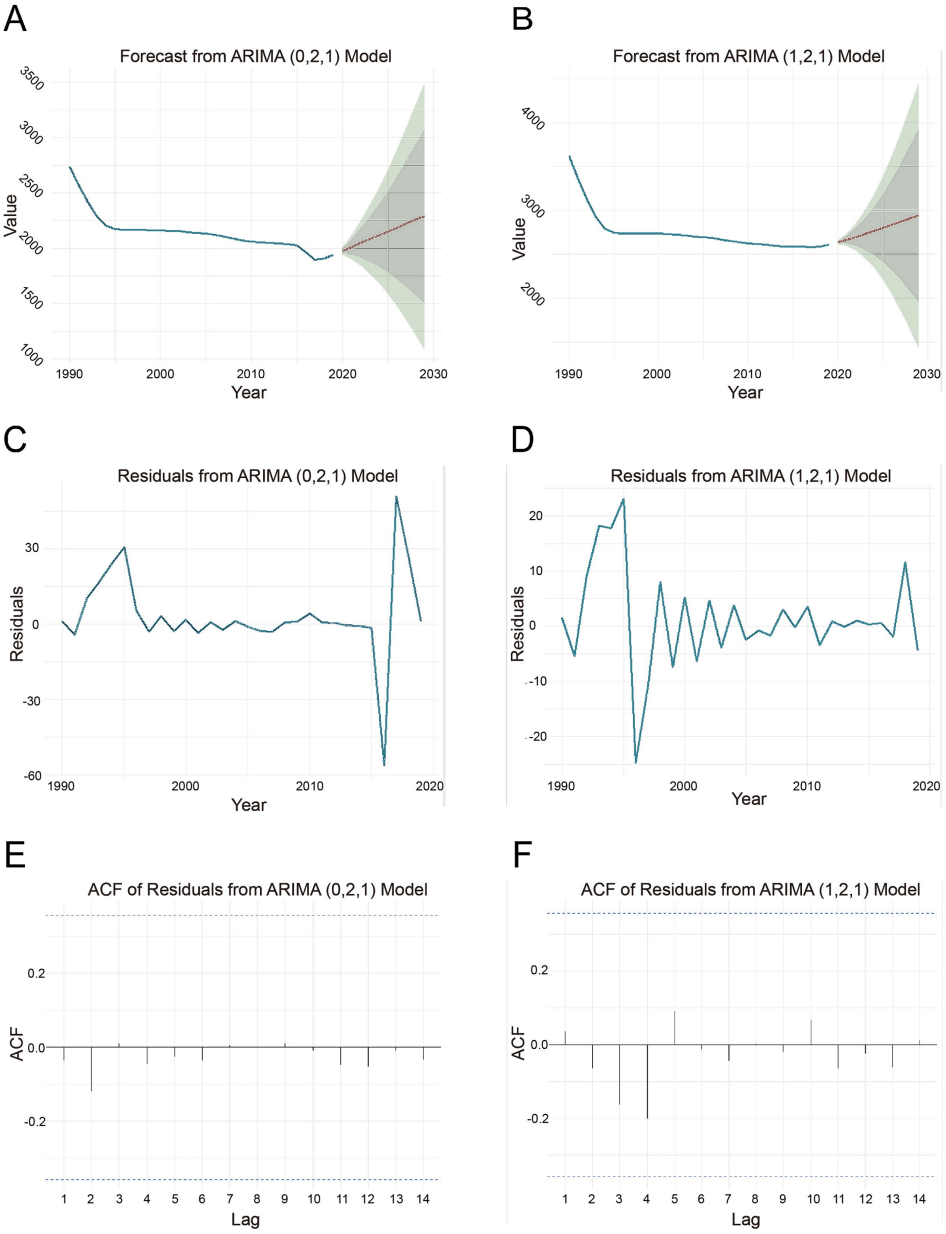
Forecast of LBP incidence rates (per 100,000) from 2020 to 2029 through ARIMA. **(A)** LBP in male subjects. **(B)** LBP in female subjects. **(C)** Residuals from ARIMA (0,2,1) model in male subjects. **(D)** Residuals from ARIMA (1, 2) model in female subjects. **(E)** ACF of residuals from ARIMA (0,2,1) model in male subjects. **(F)** ACF of residuals from ARIMA (1, 2) model in female subjects. ACF, autocorrelation function; LBP, low back pain; ARIMA, autoregressive integrated moving average.

The ADF test for LBP time series data of Chinese men indicated a significant trend (*p*-value = 0.0292), confirming the series was smooth. White noise analysis and the Ljung-Box test (*p* < 0.05) confirmed the presence of autocorrelation, suggesting the time series was not white noise. Meanwhile, most partial autocorrelation coefficients were within the CIs for all the lags, the PACF suggested that an AR (1) model might be appropriate. Filtered by auto.arima () function, ARIMA (0,2,1) was found to be the optimal model for predicting the trend of LBP incidence in men. The fitted and predicted values are shown in Figure 4A. Residual plots and ACF plots of the residuals (Figures 4C,E) showed fluctuations with outliers in specific periods (e.g., the early 1990s and the late 2010s), potentially indicating unmodelled features or events. Although these localized anomalies were observed, the residuals were close to zero overall and within CIs at all lags, indicating no significant autocorrelation and supporting the adequacy of the ARIMA (0,2,1) model. Also, the Ljung-Box Q-test results (male subjects: χ^2^ = 2.0752, df = 20, *p* = 1.00) indicated that the residual series contained white noise, supporting the model fit. Forecast results for 2020–2029 are presented in Table 4.

**Table 4:** Prediction of LBP incidence in China for the next 10 years according to ARIMA model with 95% confidence interval (per 100,000 population).

Year	Male subjects: ARIMA (0,2,1)			Female subjects: ARIMA (1,2,1)		
	Forecast	Lower limit	Upper limit	Forecast	Lower limit	Upper limit
2020	1976.66	1941.75	2011.56	2637.15	2618.04	2656.25
2021	2011.64	1904.85	2118.43	2666.06	2595.30	2736.81
2022	2046.62	1848.46	2244.79	2697.28	2542.30	2852.26
2023	2081.61	1775.77	2387.44	2730.11	2459.36	3000.86
2024	2116.59	1688.91	2544.26	2764.05	2347.85	3180.25
2025	2151.57	1589.34	2713.80	2798.76	2209.55	3387.97
2026	2186.55	1478.14	2894.97	2834.01	2046.28	3621.73
2027	2221.54	1356.18	3086.89	2869.62	1859.77	3879.48
2028	2256.52	1224.14	3288.90	2905.49	1651.60	4159.38
2029	2291.50	1082.60	3500.41	2941.54	1423.20	4459.88

LBP, low back pain; ARIMA, autoregressive integrated moving average.

The ADF test for Chinese female LBP time series indicated that the series was not smooth (*p*-value = 0.8259). After second-order differencing, the test showed a smooth time series (*p*-value = 0.01). White noise analysis showed significant autocorrelation (*p* < 0.05), suggesting that the differenced series was not white noise. PACF analysis suggested the series might be suitable for the ARIMA model. The ARIMA (1,2,1) model was identified as optimal for predicting female LBP incidence trends (Figure 4B). Residual analysis (Figures 4D,F) showed fluctuations and outliers in the early 1990s, suggesting the model may have missed some data features. However, the residuals were close to zero on all lags and were mostly within the confidence intervals with no significant autocorrelation, supporting the ARIMA (1,2,1) model. The Ljung-Box Q-test (*p* = 0.9997) confirmed the residuals contained white noise, supporting the model’s fit. Forecasts for 2020–2029 are shown in Table 4.

### 3.5 The sex specific situation of DALYs, YLDs, YLLs in China

**Figure 5:**
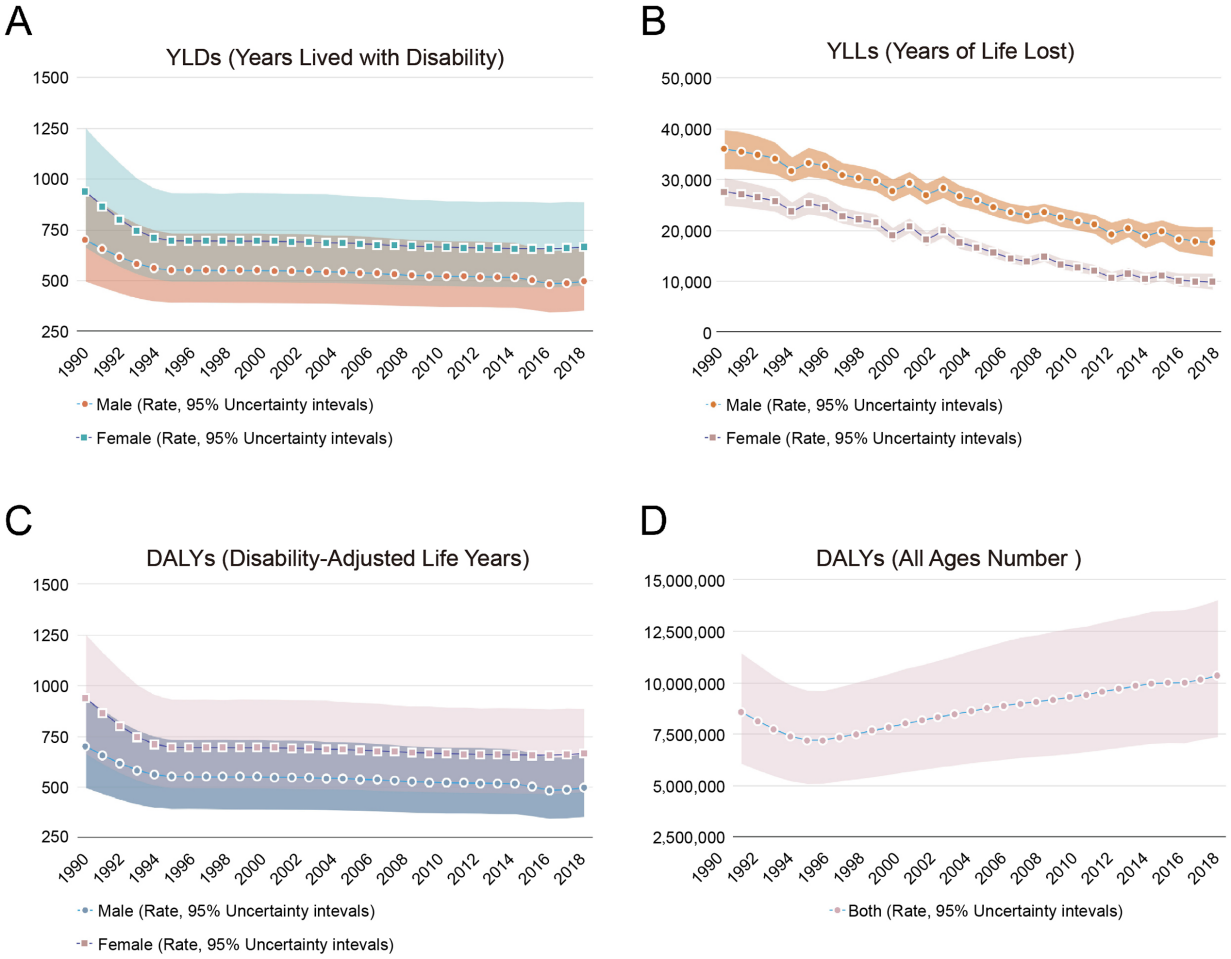
The sex specific situation of DALYs, YLDs, and YLLs in China (per 100,000) from 1990 to 2019. **(A)** Age-standardized YLDs rate. **(B)** Age-standardized YLLs rate. **(C)** Age-standardized DALYs rate. **(D)** All ages number of DALYs. DALYs, disability-adjusted life-years; YLDs, years lived with disability; YLLs, years of life lost.

Between 1990 and 2019, men experienced fewer years lost to ill health than women, though the absolute number has been increasing. YLDs represent the health lost to disease or injury, indicating the time spent in a state of poor health (15). Over the past 30 years, the age-standardized YLDs rates for both men and women were remained stable since 1995, with men consistently having lower rates than women (Figure 5A). YLLs represent the number of years lost to premature death due to disease or injury, reflecting the time not lived to life expectancy due to disease or injury (16). The level of YLLs in China has generally declined over the past 30 years, with men consistently having higher YLLs than women. Men consistently had higher YLLs than women, while the age-standardized YLDs rate for Chinese men was continuously declining. The specific data are presented in Figure 5B. DALYs, a composite indicator combining YLDs and YLLs, measure health loss due to illness or injury (15). DALYs in China declined between 1990 and 1995, remaining stable for the subsequent 25 years. The age-standardized DALYs rate for women was higher than that for men, but both have been decreasing (Figure 5C). However, similar to the YLDs data, the all ages number of DALYs also showed an upward trend (Figure 5D).

## 4 Discussion

This study provides a more comprehensive analysis of GBD LBP data, covering 30 years of LBP epidemiological data from 1990 to 2019. It not only analyzes the prevalence and incidence data using Joinpoint Regression analyses, age-period-cohort analyses, and projections for the next 10 years via the ARIMA model, but also explores potential causes from an SDI perspective. Other studies have typically focused on DALYs as a composite indicator of LBP burden (17, 18); however, separate analyses can reveal whether the disease affects longevity (via YLLs) or quality of life (via YLDs). LBP may contribute less to YLLs but more to YLDs, thus showing a stronger correlation between YLDs and DALYs, which has important implications for the development of targeted public health interventions. In this study, DALYs, YLDs, and YLLs were analyzed separately to measure health loss due to disease or injury. A detailed analysis from a gender perspective provides a clearer view of gender disparities in policymaking, thereby offering a more precise systematic analysis of LBP burden and a stronger theoretical foundation for policymakers to allocate healthcare resources, manage diseases, and design surveillance measures.

In this study, we found that the ASPR of the Chinese population generally decreased from 1990 to 2019; however, the total number of people suffering from the disease increased. This suggests that the absolute burden of LBP in China has increased to a certain extent, but its relative burden might be decreasing, which might be related to the China’s population growth and aging. ASPR account for changes in population’s age structure, reflecting actual trends within specific age groups. Excluding the influences of age structure, LBP prevalence decreased across all age group. This is consistent with previous studies on the prevalence of LBP in China (19). In our study, both age-standardized YLDs rate and DALYs rate in China remained stable after a brief decline, whereas the age-standardized YLLs rate had been at a declining level. The curves of YLDs and DALYs were coupled to a higher degree, which might indicate that LBP may contribute less to YLLs but more to YLDs, suggesting that the disease mainly affects quality of life through disability burden, and that public health strategies should focus on rehabilitation, symptom relief, and measures to improve quality of life. Although the age-standardized YLDs and DALYs rates remained stable, the total number of cases for both increased sharply, indicating that the absolute burden of LBP had been rising in China. The conclusion is generally consistent with a 2016 DALYs study on LBP in China (20), though some discrepancies in the data may be attributed to updates in the GBD data.

In this study, ARIMA modeling predicted a slight increase in LBP prevalence among women and men over the next decade, whereas prevalence in women was expected to higher than men. Despite an overall decline in the incidence and prevalence of LBP, the burden of LBP is expected to rise as the population ages, given the higher prevalence in women. This aligns with gender-specific analyses and findings from other studies (4, 21). A study on lifetime prevalence of LBP in heterosexual twins from Australia, Spain, and America found a significant gender difference only in Spain (22). This does not align with the results of our study. This may be due to the generally younger populations (mean age 47.4 years) of the participants included in this study, while peak LBP incidence occurs in older populations. Though the reasons for higher LBP prevalence in women remain unclear, possible explanations may include physiological factors such as pelvic structure, ligamentous laxity, and the menstrual cycle (3, 23, 24). Pregnancy-related weight gain and changes in the body’s center of gravity may also contribute to LBP (25, 26). These physiological factors may explain the higher LBP prevalence in women. Occupational and domestic responsibilities may also contribute. Despite changing gender roles in China, women still handle more housework, such as cleaning and childcare, which often involve bending and lifting, increasing the burden on the lower back (27). Some female-dominated occupations, such as nursing, also require long periods of standing or maintaining a fixed posture, which may increase the risk of LBP (28). Studies show that stress and emotional problems are linked to LBP, and women may experience higher levels of stress due to dual social and family roles, exacerbating symptoms (29, 30). Therefore, health resources should be allocated strategically, and targeted programs should be developed to reduce the LBP burden in women.

In our study, the parallel trends in YLLs and YLDs between genders suggest that systemic factors (e.g., healthcare policies and occupational safety reforms) impact both groups similarly, but baseline disparities remain entrenched. Targeted interventions addressing female-specific risks, such as workplace ergonomic adjustments, subsidized prenatal/postpartum care, and mental health support, may help to narrow this gap. Future research should explore longitudinal associations between gendered occupational exposures, hormonal profiles, and LBP severity to refine prevention strategies.

In our study, age-related analyses indicated that the risk of LBP increases with age, consistent with an Australian study (31). This finding suggests that the age-related pattern change in LBP with is universal. This may be linked to several factors, including degenerative changes in the spine and its surrounding structures such as intervertebral discs, joints and ligaments, increasing the risk of LBP (32–34). Decreased activity and insufficient physical activity in older adults may lead to decreased muscle strength and flexibility, increasing the risk of LBP (35). In addition, prolonged poor posture, such as sedentary lifestyle, may exacerbate LBP (36). Chronic conditions, such as diabetes, cardiovascular disease, and osteoporosis, are more common among the older adults and may directly or indirectly increase LBP risk (37–39). For example, osteoporosis may lead to vertebral compression fractures, which can cause LBP; diabetes is often associated with weight gain, which may add stress to the spine and worsen LBP. These factors should be further validated through specific data and studies. Therefore, future research should prioritize management and prevention strategies for LBP in the older and female population, especially high-risk groups. Such studies are crucial for developing health policies for the older population.

Our findings indicate that the prevalence of LBP may be higher in regions with a high SDI than in those with a low SDI. This disparity could be attributed to the interplay of multiple factors, including stress levels, gender roles, medical resources, and lifestyle. Individuals in high SDI regions are often subjected to greater work stress, social competition, and psychological burdens, which may increase the risk of LBP. The accessibility of medical resources is typically higher in high SDI regions, which may lead to a higher diagnosis rate of LBP, while the prevalence of LBP in low SDI regions may be underestimated due to insufficient medical resources. Additionally, a sedentary lifestyles and high-intensity occupational stress, common in high SDI regions, may increase the risk of LBP. In contrast, low SDI regions, where physical labor is more prevalent, may experience different types and distributions of pain.

## 5 Limitation

This study has some limitations. The GBD estimates are based on modeled data and may not fully reflect regional variations within China. Although ASRs allow comparison across years, some contextual factors (e.g., socioeconomic or lifestyle changes) were not explicitly incorporated. Estimates of DALYs, YLDs, and YLLs depend on standard GBD assumptions, and classification updates over time may influence long-term comparability. Statistical methods such as Joinpoint regression, Age–Period–Cohort analysis, and ARIMA forecasting are sensitive to data grouping and model specification, which may affect subtle trend interpretation. In addition, the GBD’s one-day case definition captures mild and transient episodes, potentially leading to higher overall burden estimates; future studies could stratify by pain severity or functional impairment for more clinically relevant assessments.

## 6 Conclusion

LBP has imposed a heavy public health burden on China over the past three decades, and this burden is poised to increase further amid the population ageing. The burden is greater among women than men. Public health interventions and clinical diagnostic and treatment protocols that account for gender differences in low back pain are of particular importance.

## Data Availability

Publicly available datasets were analyzed in this study. This data can be found here: https://www.healthdata.org/research-analysis/gbd.

## Footnotes

^1^ https://ghdx.healthdata.org/
